# Correction to: Usefulness of Fibrosis-4 (FIB-4) score and metabolic alterations in the prediction of SARS-CoV-2 severity

**DOI:** 10.1007/s11739-022-03056-z

**Published:** 2022-07-21

**Authors:** Rosa Lombardi, Vincenzo La Mura, Annalisa Cespiati, Federica Iuculano, Giordano Sigon, Giada Pallini, Marco Proietti, Irene Motta, Beatrice Montinaro, Elisa Fiorelli, Matteo Cesari, Alessandra Bandera, Luca Valenti, Flora Peyvandi, Nicola Montano, Marina Baldini, Anna Ludovica Fracanzani

**Affiliations:** 1 Unit of Internal Medicine and Metabolic Diseases, Fondazione IRCCS Ca’ Granda Ospedale Maggiore Policlinico, Milan, Italy; 2 Department of Pathophysiology and Transplantation, Università Degli Studi di Milano, Via F. SFORZA 35, 20122 Milan, Italy; 3 Unit of Internal Medicine Hemostasis and Thrombosis, Fondazione IRCCS Ca’ Granda Ospedale Maggiore Policlinico, Milan, Italy; 4 Department of Biomedical Sciences for Health, Università degli Studi di Milano, Milan, Italy; 5 Geriatric Unit, IRCCS Istituti Clinici Scientifici Maugeri, Milan, Italy; 6 Department of Clinical Sciences and Community Health, Università degli Studi di Milano, Milan, Italy; 7 Unit of Internal Medicine, Fondazione IRCCS Ca’ Granda Ospedale Maggiore Policlinico, Milan, Italy; 8 Unit of Internal Medicine and Immunology, Fondazione IRCCS Ca’ Granda Ospedale Maggiore Policlinico, Milan, Italy; 9 Unit of Infectious Diseases, Fondazione IRCCS Ca’ Granda Ospedale Maggiore Policlinico, Milan, Italy; 10 Department of Transfusion Medicine and Hematology, Università degli Studi di Milano, Milan, Italy

## Correction to: Internal and Emergency Medicine 10.1007/s11739-022-03000-1

In the original version of the article, the order of name and surnames of authors has been inverted.

The correct order should be.

Rosa Lombardi, Vincenzo La Mura, Annalisa Cespiati, Federica Iuculano, Giordano Sigon, Giada Pallini, Marco Proietti, Irene Motta, Beatrice Montinaro, Elisa Fiorelli, Matteo Cesari, Alessandra Bandera, Luca Valenti, Flora Peyvandi, Nicola Montano, Marina Baldini, Anna Ludovica Fracanzani.

The original article has been updated.

